# Where Sepsis Meets Haemorrhage: A Case Report of Spontaneous Splenic Rupture Following Perforated Diverticulitis

**DOI:** 10.1155/cris/5579279

**Published:** 2026-09-24

**Authors:** Shasha Haycock, Annie Jiao Wang, Kristy Mansour, Khang Duy Ricky Le, Andrew MacLeod

**Affiliations:** ^1^ Department of General Surgical Specialties, The Royal Melbourne Hospital, Melbourne, Victoria, Australia, thermh.org.au; ^2^ Department of General Surgery, Northeast Health Wangaratta, Wangaratta, Victoria, Australia, northeasthealth.org.au; ^3^ Department of Medicine, The University of Melbourne, Melbourne, Victoria, Australia, unimelb.edu.au; ^4^ Department of General Surgery, Western Health, Melbourne, Victoria, Australia, westernhealth.org.au

**Keywords:** case report, complicated diverticulitis, diverticulitis, rupture, splenic rupture, spontaneous

## Abstract

Atraumatic splenic rupture is an extremely rare cause of deterioration in the context of concurrent abdominal pathology. This case report describes rapid deterioration and shock in a mid‐50s‐year‐old man with acute complicated sigmoid diverticulitis 3 days into admission without history of abdominal trauma. This was associated with a rapid haemoglobin (Hb) drop from 119 to 37 g/L, which necessitated activation of the massive transfusion protocol. An atraumatic splenic rupture was subsequently diagnosed on urgent exploratory laparotomy. The patient underwent splenectomy followed by a Hartmann’s procedure for treatment of perforated sigmoid diverticulitis. This case highlights the importance of maintaining a broad differential diagnosis and consideration of concurrent pathologies for deteriorating patients with common acute infectious abdominal pathologies, including appendicitis or diverticulitis, as missed diagnosis of atraumatic splenic rupture may be fatal due to profound haemorrhage.

**Summary**

Atraumatic splenic rupture is an extremely rare cause of deterioration in the context of concurrent infective abdominal pathologies, including diverticulitis. This may occur in the absence of radiological or histological abnormalities of the spleen. Early recognition and management are crucial for this potentially fatal condition.

## 1. Background

Splenic ruptures are categorised into traumatic and atraumatic mechanisms. Atraumatic splenic rupture is a rare surgical emergency, with a 12.2% mortality rate and accounts for 1%–7% of all splenic ruptures [1]. Proposed aetiologies include infectious, neoplastic and inflammatory (93%) pathologies, with a smaller category of idiopathic cases (7%) [2, 3]. The underlying pathophysiology, however, remains poorly understood.

In patients with acute infectious abdominal pathology, atraumatic splenic rupture is rarely considered as a differential for deterioration given the rarity of the condition and the more common anticipated differential of septic shock [4, 5]. However, if unrecognised, atraumatic splenic rupture can lead to profound haemorrhagic shock and associated morbidity and mortality. Herein, we report a case of diverticulitis complicated by haemorrhagic shock secondary to atraumatic splenic rupture requiring emergency laparotomy. This case has been reported in line with the Surgical Case Report (SCARE) criteria [6].

## 2. Case Presentation

The patient provided written consent for the de‐identification and use of their medical information and data for the generation and publication of this case report.

### 2.1. Case History and Examination

A mid‐50‐year‐old male presented via personal transport to the emergency department with sudden‐onset lower abdominal pain. His past medical history included ischaemic heart disease managed with percutaneous intervention approximately 6 years prior, ongoing dual antiplatelet therapy and previous traumatic subarachnoid and subdural haemorrhages. He was haemodynamically stable and afebrile on presentation, with focal peritonism in the left iliac fossa.

### 2.2. Differential Diagnosis, Investigations and Management

**Figure 1:**
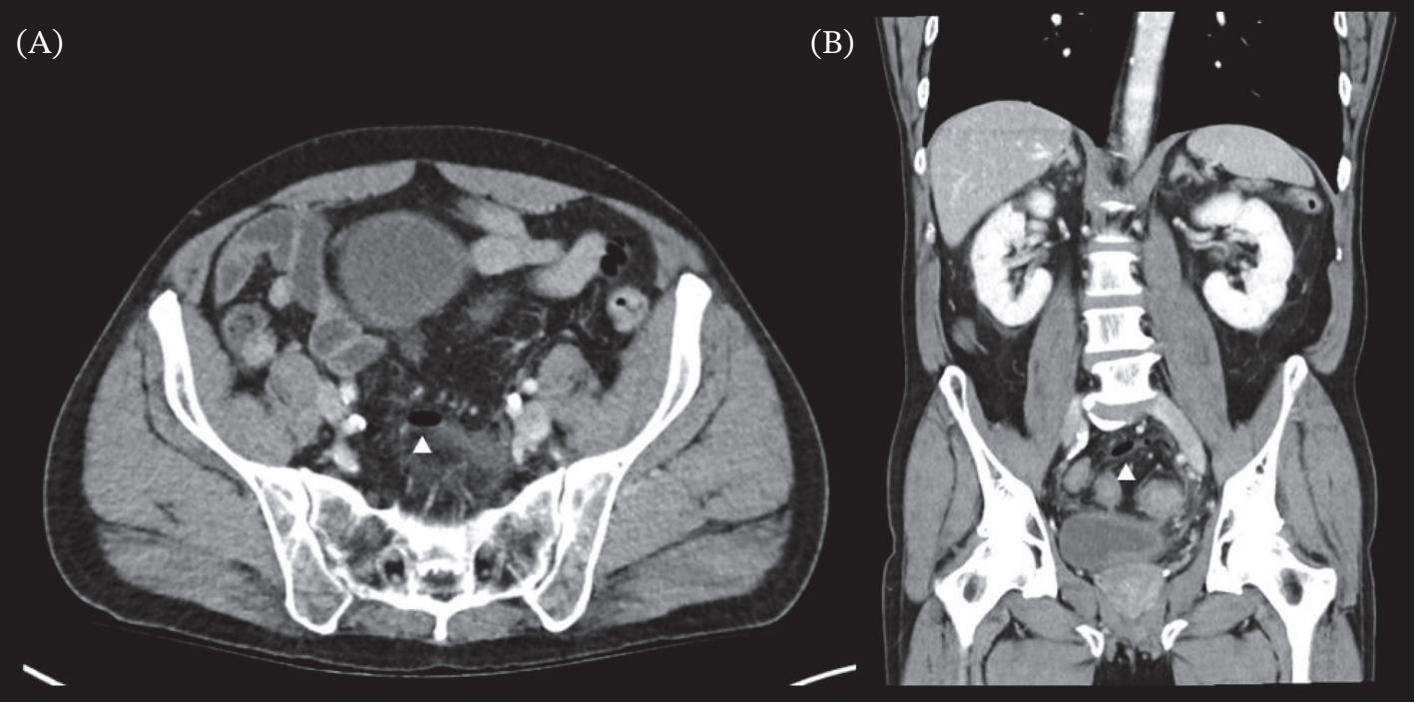
Admission computed tomography scan of the abdomen and pelvis in the portal‐venous phase demonstrating coronal (A) and axial (B) views of sigmoid diverticulitis with findings of retroperitoneal free gas and pelvic fluid collection (white arrows) consistent with a localised perforation.

A computed tomography (CT) scan of the abdomen and pelvis demonstrated sigmoid diverticulitis with local perforation, with small locules of gas adjacent to the sigmoid colon and a small pelvic collection (Figure 1). Given that the patient was afebrile with localised symptoms only, a trial of non‐operative management was considered reasonable. The patient was consequently managed non‐operatively with intravenous antibiotics (piperacillin and tazobactam at a dose of 4.5 g every 6 h) and serial examination. He demonstrated clinical and biochemical improvement during the first 2 days of admission.

**Figure 2:**
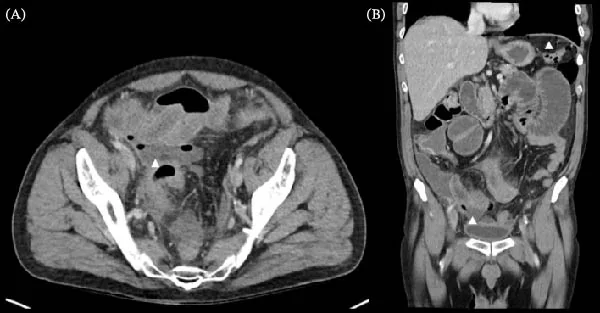
Repeat computed tomography scan of the abdomen and pelvis in the portal‐venous phase performed on day 3 of admission demonstrating coronal (A) and axial (B) views of possible small bowel obstruction with free gas and increasing pelvic collection (white arrows).

On day three of admission, the patient had worsened abdominal pain, with signs of increasing abdominal distention and rising inflammatory markers. A repeat CT scan of the abdomen and pelvis demonstrated increasing size of his known pelvic collection, increased free gas and potential new adhesive small bowel obstruction (Figure 2). Further rapid deterioration resulted in a Medical Emergency Team (MET) call for hypotension (systolic blood pressure 65/56 mmHg), during which he received fluid resuscitation for differentials including hypovolaemia or sepsis. He remained hypotensive with a SBP around 90 mmHg despite 3 litres of resuscitation with crystalloid and albumin. Biochemical examination at this time revealed a pH of 7.1 (normal range 7.35–7.45), lactate of 17 mmol/L (normal range <2 mmol/L), base excess −19.9 (normal range −2 to +2 mmol/L) and mild AKI with a creatinine of 51 progressing to 117 mmol/L (normal range 60–110 µmol/L). There was further rapid clinical deterioration, manifesting as hypothermia, diaphoresis, and peripheral pallor. Given concerns for intra‐abdominal sepsis and shock secondary to perforated sigmoid diverticulitis, the patient proceeded to emergent laparotomy. During transfer to the operating theatre, the patient further deteriorated, becoming unresponsive and peri‐arrest, with a Glasgow Coma Scale score of 3. Pre‐operative venous blood gas at this time demonstrated a haemoglobin (Hb) of 37 g/L (normal range 135–170 g/L for an adult male), decrementing from 119 g/L 7 h. This raised clinical suspicion for acute active haemorrhage and prompted activation of the massive transfusion protocol. On exploratory laparotomy, large‐volume haemoperitoneum was identified secondary to a ruptured spleen. Additional findings included small bowel obstruction secondary to inflammatory adhesions in the pelvis and perforated sigmoid diverticulitis with associated pelvic abscess. Damage control principles were employed, with the patient undergoing splenectomy, followed by Hartmann’s procedure for both haemorrhagic and septic source control. The abdomen was closed with temporary VAC laparostomy, and post‐operatively, the patient was transferred to the intensive care unit (ICU) for ongoing resuscitation with a view to re‐look laparotomy at 48 h after the index surgery.

### 2.3. Follow‐Up Outcomes

Two days post index operation, a re‐look laparotomy, adhesiolysis, formation of end‐colostomy and closure were performed, finding no further intra‐abdominal pathology. The patient returned to the ICU and was extubated 1 day later, commenced on feeds day three post closure and discharged to the ward day five. The patient had an uncomplicated post‐operative course and was discharged home day nine post relook laparotomy and closure. As per Spleen Australia guidelines, the patient had spleen prophylaxis vaccinations, including the pneumococcus conjugate vaccine, meningococcus conjugate ACWY and MenB vaccies, the hameophilus influenzae type B conjugate Hb vaccine and the influenza vaccine [7]. The patient was also commenced on daily oral amoxicillin 250 mg with an emergency script provided (2 g to four 500 mg capsules) and educated to utilise this supply if any systemic features of sepsis were experienced. At the time of post‐operative follow‐up 2 weeks following his initial operation, the patient was well without features of any infection or bleeding and planned for later follow‐up with the surgeon to consider stoma reversal.

Histopathology of a 90 × 50 × 60 mm spleen weighing 148 g demonstrated one of the two sections of splenic tissue with haemorrhagic changes with subcapsular haematoma and a disrupted splenic capsule; the splenic tissue was otherwise unremarkable. Microscopically, the spleen demonstrated evidence of focal extramedullary haematopoiesis without any evidence of malignancy. The sigmoid colon demonstrated diverticulitis with florid organising acute serositis, consistent with diverticular rupture. Retrospective review of the two preoperative CT scans also did not demonstrate any abnormality within the spleen within the limitations of being a single‐phase portal venous scan.

## 3. Discussion

Our case represents the third reported case of spontaneous splenic rupture secondary to intraabdominal sepsis, with only two previous case studies identified within the past decade [4, 5]. It highlights the need for clinicians to be aware of the atraumatic ruptured spleen as a potential concurrent life‐threatening pathology in the deteriorating patient being treated for intra‐abdominal pathology. Challenges highlighted in this case include early identification of a rapidly deteriorating patient with this condition to facilitate urgent definitive operative management as delays in recognition may ultimately prove fatal.

As described by Park et al., a patient’s sudden deterioration may be mistaken as septic and distributive shock rather than haemorrhagic shock [5]. Our case similarly demonstrates the difficulty of differentiating the two aetiologies of shock and highlights that these two pathologies may progress in parallel. In fact, it is difficult to identify in the present case study, the timing of the spontaneous splenic rupture. The repeat CT scan on day three of admission notes the absence of splenic haemorrhage at the time of initial clinical deterioration. This suggests that the patient’s initial symptoms likely relate to the progression of perforated diverticular disease despite conservative management. What remains unclear is if the medical escalation overnight prior to laparotomy represents an early contained and partially compensated splenic haemorrhage versus progressive septic shock, which was temporarily responsive to fluid resuscitation. Regardless, the subsequent persistent instability followed by rapid deterioration to a peri‐arrest state, with marked acute Hb decline, led to immediate activation of the massive transfusion protocol and prompt procession to urgent exploratory laparotomy with the finding of an unexpected splenic rupture.

The management of atraumatic splenic rupture follows the general ABCDE principles of resuscitation, including securing the airway, correcting hypoxaemia, venous access for early resuscitative fluids/blood products, antibiotics and correction of hypothermia [8]. If haemorrhagic shock is suspected, early activation of a massive transfusion protocol is recommended. Goal‐directed fluid resuscitation should take into consideration haemodynamic status, co‐morbidities, biochemical derangements and time to definitive management as highlighted in the present case.

The pathogenesis of spontaneous splenic rupture is poorly understood due to the rarity of the condition, with a paucity of literature on the topic. One proposed mechanism describes oedema, cellular hyperplasia and neutrophil infiltration resulting in increased trans‐splenic pressure and subsequent parenchymal or subcapsular haematoma and capsular tear [9, 10]. Potential drivers include splenic thrombosis, infarction and shearing forces caused by diaphragmatic or abdominal muscle contraction secondary to coughing [11, 12]. In our case, histological examination of the spleen and its hilum was normal without oedema, cellular hyperplasia, increased neutrophilia or thrombosis to support these theories. Furthermore, preoperative CT did not demonstrate any anatomical abnormalities of the spleen. More broadly, atraumatic causes of splenic rupture can be classified as infectious and non‐infectious, with the distributions of these entities varying depending on geographical distribution. Infectious causes may include viral illnesses, including Epstein‐Barr Virus (EBV), cytomegalovirus (CMV) and human immunodeficiency virus (HIV), bacterial infection including typhoid fever and in endemic regions, malaria infections. The latter is important to note as splenic rupture has been reported in the literature to be a recognised and potential life‐threatening complication of Plasmodium knowlesi infection and therefore should be considered as a differential diagnosis for the acute atraumatic spleen rupture in patients with a travel history to endemic areas [13]. Non‐infectious causes, particularly in our case where these endemic infections are less common, include haematological malignancies and inflammatory disorders (such as acute pancreatitis).

## 4. Conclusion

Our case highlights the need to consider atraumatic splenic rupture as a rare cause of shock with concurrent intra‐abdominal sepsis. Early recognition may facilitate prompt diagnosis and management of a potentially fatal condition. The pathogenesis of this condition remains poorly understood and requires further research to elicit; however, we note that atraumatic splenic rupture may occur in the absence of radiological or histological abnormalities of the spleen.

## Author Contributions

**Shasha Haycock:** conceptualisation, data curation, methodology, formal analysis, resources, writing – original draft, writing – review and editing. **Annie Jiao Wang:** data curation, methodology, resources, writing – original draft, writing – review and editing. **Kristy Mansour:** conceptualisation, data curation, methodology, resources, writing – review and editing. **Khang Duy Ricky Le:** data curation, methodology, resources, writing – original draft, writing – review and editing. **Andrew MacLeod:** conceptualisation, writing – review and editing, supervision.

## Funding

No funding was received for this manuscript.

## Ethics Statement

The case report generation process was discussed with our local ethics and governance team. No formal ethics approval was required following the discussions and therefore was waived. The patient provided consent for the de‐identification and use of their medical information and data for the generation and publication of this case report.

## Consent

The patient provided informed consent that was written and signed for the generation and publication of this manuscript using their de‐identified medical information.

## Conflicts of Interest

The authors declare no conflicts of interest.

## Data Availability

Data can be requested from the corresponding author when required. All relevant data has been provided in the generation of this manuscript which is intended for open access publication.
